# Actomyosin contractility controls cell surface area of oligodendrocytes

**DOI:** 10.1186/1471-2121-10-71

**Published:** 2009-09-25

**Authors:** Angelika Kippert, Dirk Fitzner, Jonne Helenius, Mikael Simons

**Affiliations:** 1 Max-Planck-Institute for Experimental Medicine, Hermann-Rein-Str. 3, Göttingen, Germany; 2 Department of Neurology, Robert-Koch-Str. 40, University of Göttingen, Göttingen, Germany; 3 Deptartment of Cellular Machines, Biotechnology Center, University of Technology, Tatzberg 47-51, Dresden, Germany

## Abstract

**Background:**

To form myelin oligodendrocytes expand and wrap their plasma membrane multiple times around an axon. How is this expansion controlled?

**Results:**

Here we show that cell surface area depends on actomyosin contractility and is regulated by physical properties of the supporting matrix. Moreover, we find that chondroitin sulfate proteoglycans (CSPG), molecules associated with non-permissive growth properties within the central nervous system (CNS), block cell surface spreading. Most importantly, the inhibitory effects of CSPG on plasma membrane extension were completely prevented by treatment with inhibitors of actomyosin contractility and by RNAi mediated knockdown of myosin II. In addition, we found that reductions of plasma membrane area were accompanied by changes in the rate of fluid-phase endocytosis.

**Conclusion:**

In summary, our results establish a novel connection between endocytosis, cell surface extension and actomyosin contractility. These findings open up new possibilities of how to promote the morphological differentiation of oligodendrocytes in a non-permissive growth environment.

See related minireview by Bauer and ffrench-Constant:

## Background

During their life cycle many cells undergo dramatic changes in their cell morphology including rapid alterations in shape and surface area. One way by which cells change their surface area is to alter membrane traffic between surface and interior. For example, loss of integrin-mediated adhesion during tumor cell growth triggers the rapid caveolin-mediated endocytosis that leads to cell rounding [1-3]. Another example is the cell surface area change that occurs during mitosis. At the onset of mitosis the mother cell starts to round up, and subsequently recovers when entering into anaphase. These changes of plasma membrane area are regulated by changes in endosomal membrane traffic [4].

A striking example of cell-shape change is the morphological differentiation of oligodendrocytes during the development of the central nervous system. First, small and bipolar oligodendrocyte precursor cells (OPCs) migrate into the brain, where they differentiate into oligodendrocytes and expand their plasma membrane many folds in order to wrap it several times around an axon [5,6]. In this phase of myelination, each oligodendrocyte produces up to ~50 × 10^3 ^μm^2 ^of myelin surface membrane per day [7].

Since the generation of myelin occurs at the appropriate time during neuronal development, it is assumed that neuron-glia communication coordinates this process. However, in cell culture oligodendrocytes differentiate and extend large myelin membrane sheets in the absence of neurons [8] indicating that the default action of oligodendrocytes is to differentiate. It is likely that in vivo many different positive and negative signals from other cells and the extracellular environment are integrated to control proper and timely myelination. Previous studies highlight the importance of extracellular matrix/integrin interactions for the regulation of myelin-membrane sheet extension [9]. Other studies have shown that the inactivation of the small Rho-GTPase, Rho A, is required for the morphological differentiation of oligodendrocytes [10]. In addition, changes in membrane trafficking accompany the growth of oligodendroglial cell surface area [11-14].

In this study, we tested how the extracellular environment controls cell shape and membrane traffic in oligodendroglial cells.

## Methods

### Antibodies

The following primary antibodies were used: actin (mouse monoclonal IgG, Sigma, St. Louis, MO), O1 (mouse monoclonal IgM), MBP (mouse monoclonal IgG; Sternberger Inc., Lutherville, MD), nonmuscle myosin II heavy chain A and B (polyconal Covance, Berkely, CA) and betaIII-Tubulin (mouse monoclonal IgG, Sigma, St. Louis, MO). Secondary antibodies were purchased from Dianova (Hamburg, Germany).

### Preparation of acrylamid gels on glass coverslips

To prepare acrylamid gels on glass coverslips and crosslink adhesion proteins to the gels, we adapted the protocol by [15]. Briefly, 18-mm-diameter glass coverslips were incubated overnight in 0.1 N NaOH solution, washed once with de-ionized water and dried on parafilm. Dry coverslips were covered with 100 μl of 3-APTMS (3-aminopropyltrimethoxysilane, Sigma, St. Louis, MO) for 3 min and thoroughly rinsed with de-ionized water. Glass coverslips were then incubated in 0.5% glutaraldehyd (Sigma, St. Louis, MO) for 20 min at RT followed by rinsing in de-ionized water. Coverslips were placed on parafilm with the 3-APTMS coated side on top and dried. Polyacrylamid gels were prepared by diluting 40% acrylamid 2K (AppliChem, Darmstadt, Germany) and 2% bis-acrylamid 2K (AppliChem, Darmstadt, Germany) to final concentrations of 7.5% acrylamid and 0.01% to 0.75% bis-acrylamid in PBS pH 7.4. The polymerisation was induced by adding 1.5 μl TEMED and 5 μl 10% ammonium persulfate to 1000 μl of polyacrylamid solution. 30 μl of polyacrylamid solution was pipetted onto the centre of the 18-mm-diameter 3-APTMS-coated coverslips and a 12-mm-diameter glass coverslip was placed on top of the acrylamid solution. To allow complete polymerization the gels were left at RT for 30 min before the top coverslip was removed.

### Crosslinking of adhesion proteins on polyacrylamid gels

To prevent polyacrylamid gels from drying, they were covered with 150 μl 1 mg/ml sulfo-SANPAH (Pierce, Rockford, IL) immediately after polymerisation. The gels were then places under an ultraviolet lamp at approximately 10 cm distance and irradiated for 10 min. The polyacrylamid gels were immersed in 1 ml sterile 200 mM HEPES pH 8.6 in multi well plates (12 well, Cellstar, Greiner Bio-One). The HEPES solution was aspirated and 1 ml 70% Ethanol was added for 1 min to sterilize the polyacrylamid gels. To remove residual ethanol and sulfo-SANPAH the gels were washed overnight in 1 ml sterile 200 mM HEPES pH 8.6. Coating with poly-L-lysine was done for 1 h at RT. The gels were rinsed once with de-ionized water and dried for 10 min before plating with cells.

### Cell culture and immunofluorescence

The oligodendroglial cell line, Oli-neu, was cultured on poly-L-lysine as described previously [16]. Alternatively, coverslips were coated with human fibronectin 10 μg/ml (Millipore) overnight at +4°C. Primary cultures of oligodendrocytes were obtained from newborn mice and cultured as described previously [17]. In brief, cells were plated in MEM containing B27 supplement, 1% horse serum, L-thyroxine, tri-iodo-thyronine, glucose, glutamine, gentamycine, pyruvate, and bicarbonate on poly-L-lysine coated glass coverslips after shaking. For delivery of recombinant C3 transferase (Cytoskeleton, Denver, CO) Chariot reagent (Active Motif, Carlsbad, CA) was used according to the manufacturer's protocol and cells were cultured for 10 h after protein transfection. Alternatively, cells were treated with 10 μM of ROCK inhibitor Y27632 (Calbiochem, Merck, Darmstadt, Germany) or 50 μM of blebbistatin (Sigma Aldrich, Germany) for 10 h at 37°C.

The effects of RGD-binding integrins were assessed by treating Oli-neu cells for 8 h with 100 nM RGD-peptide (#05231701, Calbiochem) or with the inactive control RAD-peptide (#03340052, Calbiochem). Poly-L-lysine pre-coated glass coverslips were incubated overnight at RT in 10 ng/ml to 500 ng/ml Chondroitin sulfate proteoglycans (CSPGs) (Proteoglycan Mix, Millipore, Billerica, MA), dried and rinsed with PBS before plating with cells. Cells were plated in a density of 50 000 per 12 well (3.8 cm^2^).

### siRNA nucleofection

Smart pool siRNA targeting myosin II-A, myosin II-B (directed at the mouse sequences (accession numbers NM_181327 and NM_175260, respectively) and of control non-targeting siRNA pool were purchased from Dharmacon (Dharmacon RNA Technologies, Lafayette, CO). Electroporation was performed using the basic neuron transfection Kit (Amaxa Biosystems, Cologne, Germany) and Amaxa programm O-05 (Amaxa Biosystems, Cologne, Germany) according to the manufacturer's protocol. Briefly, per transfection Oli-neu cells (2 × 10^6^) were harvested, resuspended in 100 μl resuspension buffer and mixed with 160 pmol siRNA per transfection. The transfection was repeated after 72 h. For electroporation of primary oligodendrocytes, 1.5 × 10^6 ^cells were collected, nucleofected with both myosin II-A and II-B or control siRNA and subsequently cultured for 3 d.

Cells were plated on coverslips and analysed for surface extension and dextran uptake after another 24 h. Knock down efficiency was controlled by western blot analysis 24 h or 48 h after the second transfection.

### Endocytosis assays

Endocytosis was assessed by incubating Oli-neu cells for 15 or 30 min at 37°C with rhodamin-labelled Dextran 10K (Molecular Probes) in culture medium. Cells were subsequently washed and fixed, followed by microscopic analysis. Image processing and analysis was performed using Meta Imaging Series 6.1 software (Universal Imaging Corporation, West Chester, PA). For the quantification of fluorescence intensities of internalized dextran a fixed threshold was applied for all images. Immunofluorescence was performed as described previously [11]. For surface labelling with Alexa Fluor 488 conjugated wheat germ agglutinin (WGA; Probes, Invitrogen, Carlsbad, CA) fixed cells were incubated with WGA for 10 min at RT, washed and mounted for microscopic analysis.

### Microscopy and analysis

Fluorescence images were acquired using a Leica (Nussloch, Germany) DMRXA microscope and a confocal laser scanning microscope (TCS SP equipped with AOBS, Leica) with 40× and 63× oil plan-apochromat objective (Leica), respecively. Image processing and analysis was performed using Meta Imaging Series 6.1 software (Universal Imaging Corporation). Quantification of fluorescence intensities was performed as described previously [11]. The cell surface area of individual cells was approximated by measuring the projected area, the area covered by the cell projected onto a single optical plane. The cells analyzed were from at least 2 independent experiments. Statistical differences were determined with Student's t test.

### Gel elasticity measurements

An atomic force microscope (AFM; NanoWizard, JPK Instruments), mounted on a Zeiss Axiovert 200 M (Carl Zeiss) was used to perform elasticity measurement. 5 micron diameter silica beads (Kisker) were attached to tip-less cantilevers (MLCT, Veeco) using epoxy glue (Araldit). Cantilever spring constants were determined using the equipartition theorem. Polyacrylamide gels were probed with a maximum indentation force of 1 nN at no less than 5 locations. Each indentation curve was fitted (Igor Pro, Wavemetrics) according to the Hertz model [18] to obtain the elasticity at each point.

## Results

### Regulation of cell surface area by the integrin binding RGD-peptide

**Figure 1:**
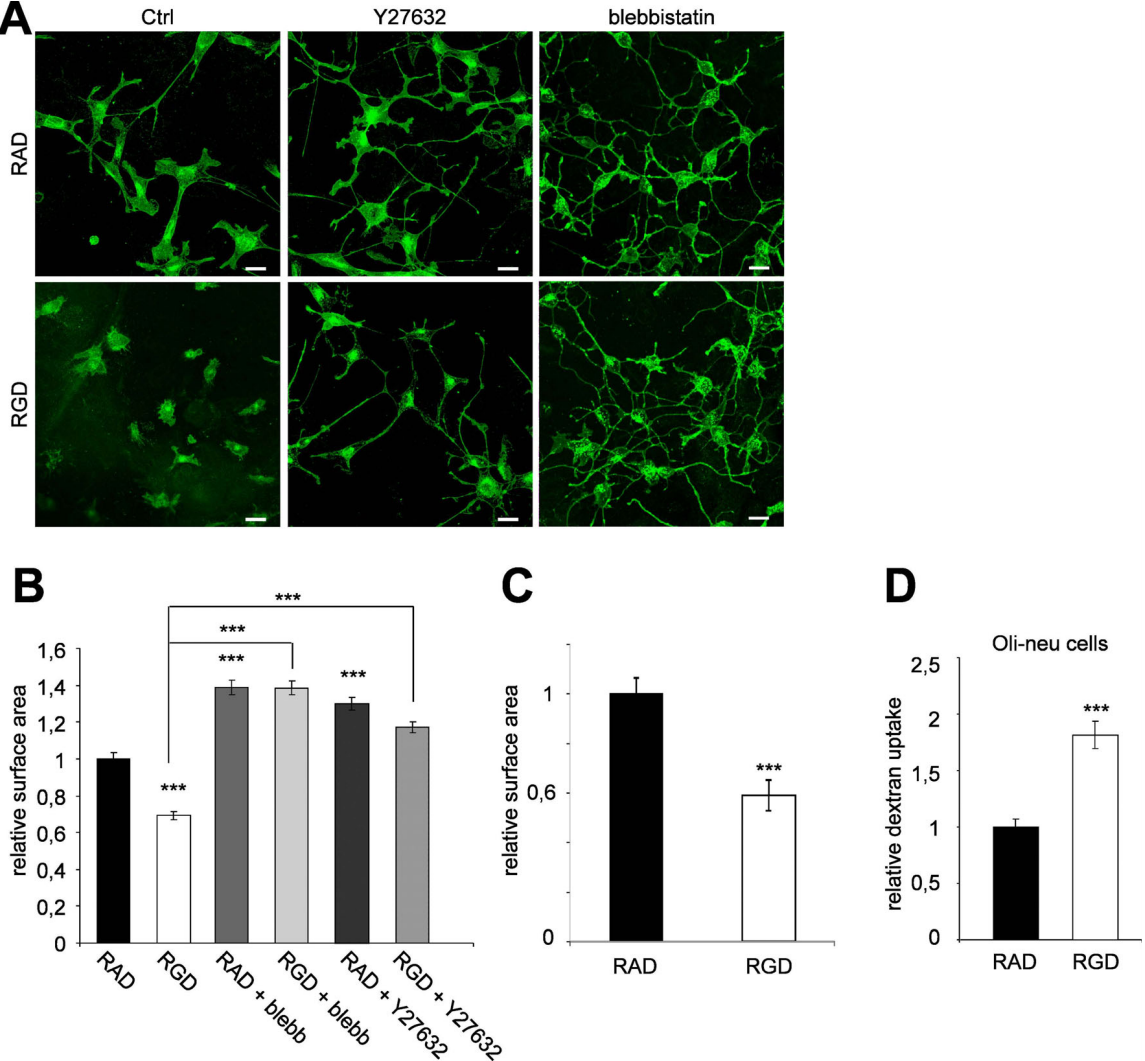
**Integrins in cell surface expansion and endocytosis in Oli-neu cells**. (A) Oli-neu cells were treated for 8 h with 100 nM RGD-peptide, 100 nM inactive RAD-peptide, 10 μM Y27632 and 50 μM blebbistatin (blebb) as indicated. Cells were stained with Alexa Fluor 488 conjugated wheat germ agglutinin (green). Scale bar, 10 μm. (B) Changes in relative surface area were quantified by image analysis as described in Material and Methods. (C) Changes in relative surface area shown for cells grown on fibronectin. (D) Quantitative analysis of dextran uptake (30 min) in Oli-neu cells treated with 100 nM RGD-peptide or inactive RAD-peptide for 8 h. Values represent means ± SEM (n > 70 cells, **p < 0,01; ***p < 0,001).

We started assessing the influence of integrins on cell surface area by treating the oligodendroglial cell line, Oli-neu, with a monovalent integrin ligand (RGD-peptide) or a non-binding control (RAD-peptide). Morphological analysis of Oli-neu cells grown on fibronectin or poly-L-lysine coated coverslips revealed a significant reduction in cell surface area after treatment with RGD-peptide compared to the inactive RAD-peptide (Figure 1A-C). Previous results suggested that changes in the rate of endocytosis control the extension of plasma membrane in Oli-neu cells [12,13]. To elucidate whether the reduction of surface area after the treatment with RGD-peptide was accompanied by changes in the rate of endocytosis, we analyzed fluid-phase endocytosis in Oli-neu cells, by studying the uptake of dextran. We found that treatment with RGD-peptide resulted in a marked increase in fluid-phase endocytosis (Figure 1D).

Signals from integrins to the Rho family GTPases regulates morphological differentiation of oligodendrocytes [10]. We speculated that the contractile forces of the actomyosin cytoskeleton areimportant for the effect of the RGD-peptide on oligodendrocytes. To test this hypothesis Oli-neu cells were treated with inhibitors of the Rho/Rho-kinase (ROCK) pathway (Y27632) and myosin II (blebbistatin) blocking actomyosin contractility. We found that treatment with either Y27632 or blebbistatin resulted in a strong increase in cell surface area (Figure 1A, B). Most importantly, the inhibitory effect of the RGD-peptide on the cell size was completely abolished in cells treated with blebbistatin and to a large extent in cells treated with Y27632 suggesting that the RGD-peptide acts by influencing actomyosin contractility. Taken together, these experiments provide evidence that integrins are involved in cell surface area control through a pathway that is dependent on contractile forces within the cells.

### Matrix rigidity and actomyosin contractility control cell surface area

There are number of recent studies demonstrating that cells sense and response to the stiffness of their substrates [19].

For example, matrix elasticity directs mesenchymal stem cell lineage specification [20]. In addition, the branching morphogenesis of neurons is modulated by physical parameters in the microenvironment [21].

**Figure 2:**
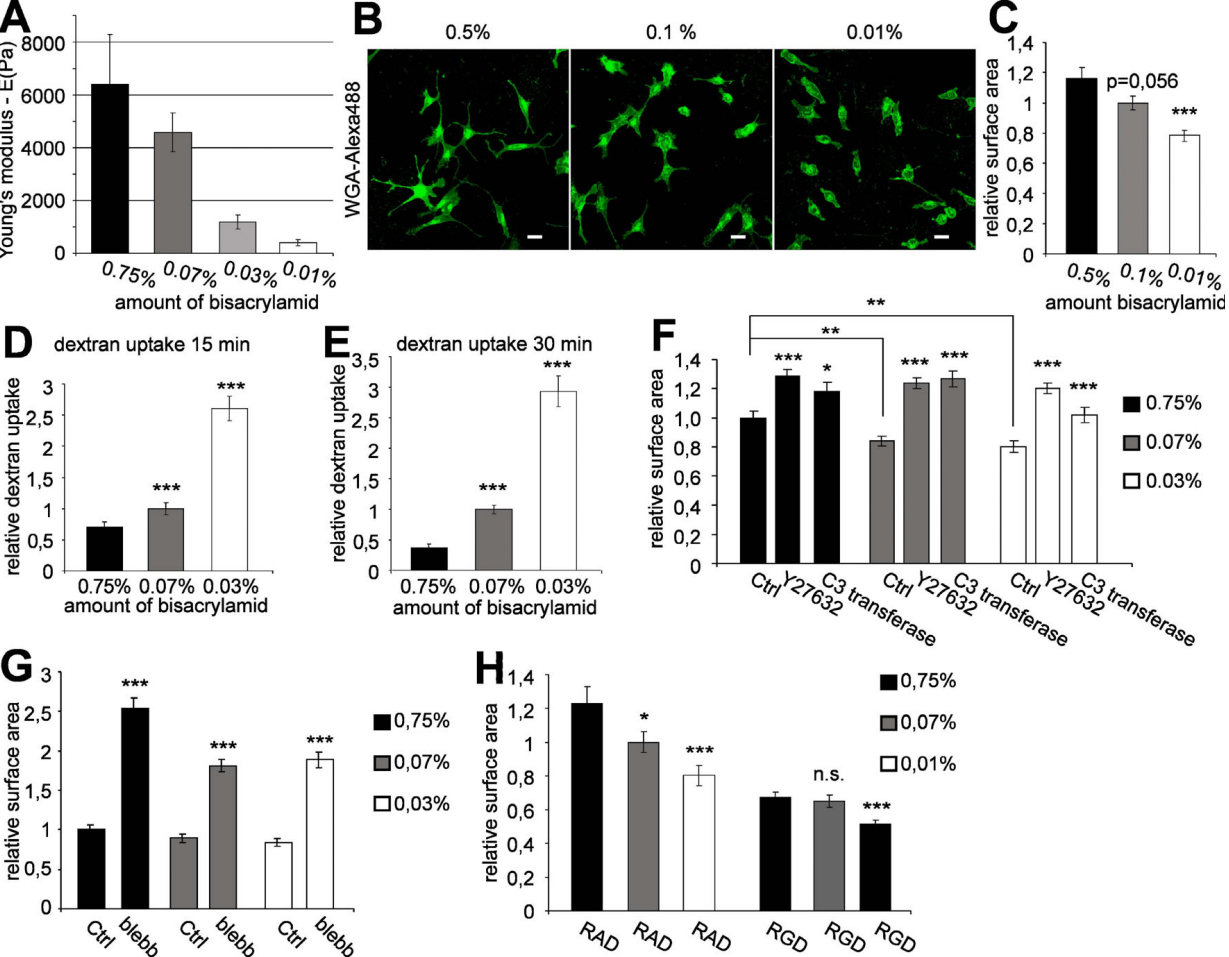
**Matrix rigidity regulates cell surface area and endocytosis in Oli-neu cells**. (A) Quantification of the elastic shear moduli of polyacrylamid gels on glass coverslips with varying amounts of bisacrylamid. Values represent means ± SD (n > 30 measurements). (B) Oli-neu cells were cultured for 1 d on polyacrylamid gels of different rigidities (using bisacrylamid varying from 0,5% to 0,01%) and analyzed for surface area differences by staining with Alexa Flour 488 conjugated wheat germ agglutinin (green). Scale bar, 10 μm. (C) Quantification of the surface area of Oli-neu cells on polyacrylamid gels of different rigidities (mean ± SEM; n > 100 cells, ***p < 0,001). (D+E) Cells were cultured for 1 d on polyacrylamid gels of different rigidities by varying the amount of bisacrylamid. Changes in the amount of dextran uptake ((D) 15 min and (E) 30 min) were quantified. Values represent the mean ± SEM (n > 60 cells from three independent experiments, ***p < 0,001). (F) Quantification of cell surface area of Oli-neu cells cultured on polyacrylamid gels for 1 d and treated for 10 h with 10 μM Y27632 or C3 transferase (mean ± SEM; n > 100 cells, *p < 0,05, **p < 0,01, ***p < 0,001). (G) Quantitative analysis of surface area changes of Oli-neu cells cultured for 1 d on polyacrylamid gels and treated with 50 μM blebbistatin (blebb) for 10 h (mean ± SEM; n > 60 cells, ***p < 0,001). (H) Oli-neu cells were cultured on polyacrylamid gels of different rigidities, treated with RGD-peptide or inactive RAD-peptide and changes in relative surface area were quantified. Values represent the mean ± SEM (n > 80 cells, *p < 0,05, **p < 0,01, ***p < 0,001).

Since integrins in conjunction with actomyosin contractility are implicated in the sensing of these physical matrix properties [22], we examined the influence of matrix properties on cell surface expansion. The use of polyacrylamid gels with variable amounts of crosslinking bisacrylamid and covalently attached poly-L-lysine allowed the physical properties of the matrix to be modulated [21,15,23]. To ensure the use of matrices of physiological rigidity their elastic modulus was measured by atomic force microscopy. The values measured for the different polyacrylamid gels varied within a physiological range (Figure 2A). When Oli-neu cells were cultured on different matrices, we observed a decrease in cell surface area on soft matrices (Figure 2B, C). As previous results indicated a connection between cell surface area and the rate of endocytosis, we investigated the effects of matrix rigidity on fluid phase endocytosis by measuring the uptake of dextran. We found that the endocytosis of dextran increased with decreasing matrix rigidity (Figure 2D, E), again suggesting a relation between cell surface area and fluid-phase endocytosis.

Next, we tested whether the inhibition of actomyosin contractility influences the effects of matrix rigidity on cell surface area. We observed that the inhibition of either Rho/ROCK signaling by C3 transferase and Y27632 or myosin II by blebbistatin restored cell surface expansion on soft matrices (Figure 2F, G). When Oli-neu cells were cultured on acrylamid gels in the presence of the RGD-peptide, the cell surface area was reduced independent of matrix rigidity, suggesting that integrins were required to sense the physical properties of the matrix (Figure 2H). However, after treatments with the RGD-peptide the cell surface area was reduced to a larger extent on the softest matrix (0.01% gels) as compared to matrices of medium (0.07%) or harder (0,75%) consistence, suggesting additional integrin-independent mechanisms in the control of membrane spreading.

By measuring the elastic modules of acrylamide gels with atomic force microscopy, we ruled out that the RGD-peptide affected matrix rigidity (the elastic module of 0.75% gels was 7761 +/- 2200 in the presence of the RGD-peptide).

**Figure 3:**
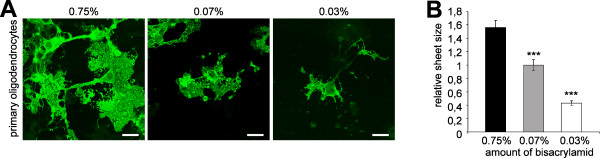
**Matrix rigidity regulates cell surface area in primary oligodendrocytes**. (A) Primary oligodendrocytes were cultured for 3-4 d on polyacrylamid gels of different rigidities and analyzed for the expansion of myelin membrane sheets by staining for MBP (green). Scale bar, 20 μm. (B) The relative sheet size of primary oligodendrocytes was quantified. Values represent the mean ± SEM (n > 60 cells, ***p < 0,001).

Next, we investigated the effects of matrix rigidity on membrane sheet expansion in primary oligodendrocytes. Again, a considerable decrease in sheet size accompanied decreasing matrix rigidity (Figure 3A, B).

### Overcoming chondroitin sulfate proteoglycans inhibition of cell surface spreading by myosin II inhibition

One group of molecules that contribute to the non-permissive growth properties in the CNS environment are chondroitin sulfate proteoglycans (CSPG) [24]. Studies have implicated a role of the Rho kinase (ROCK) pathway in CSPGs-mediated inhibition of neurite outgrowth [25].

**Figure 4:**
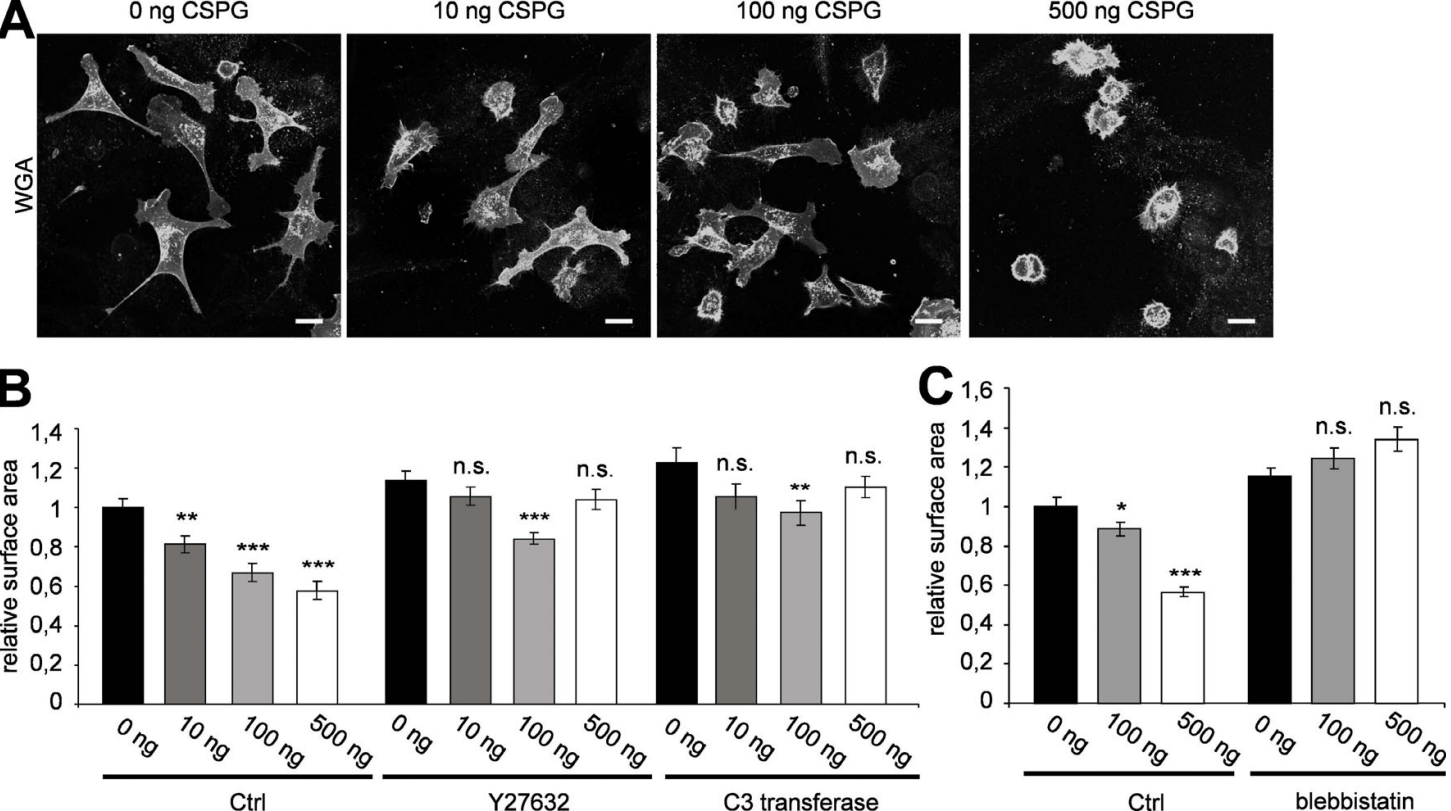
**Chondroitin sulfate proteoglycans reduce cell surface area in Oli-neu cells**. (A) Oli-neu cells were cultured for 12 h on coverslips coated with different concentrations of chondroitin sulfate proteoglycans (CSPG), stained with Alexa Fluor 488 conjugated wheat germ agglutinin (WGA) and analyzed for changes in cell surface area. Scale bar, 10 μm. (B) Quantification of the surface area of Oli-neu cells cultured on CSPG coated coverslips with or without 10 μM Y27632, C3 transferase or (C) 50 μM blebbistatin (means ± SEM; n > 80 cells, *p < 0,05, **p < 0,01, ***p < 0,001).

We, therefore, determined the effects of CSPG on the surface area of Oli-neu cells by plating the cells on different concentrations of CSPG. We observed that the cell surface area decreased with increasing concentration of CSPG. The highest concentration of 500 ng CSPG resulted round cells with diameters half that of control cells (Figure 4A, B). Quantitative analysis revealed that in the presence of Y27632, C3 transferase or blebbistatin the cell surface area of Oli-neu cells did not change at CSPG concentrations of up to 500 ng (Figure 4B, C).

**Figure 5:**
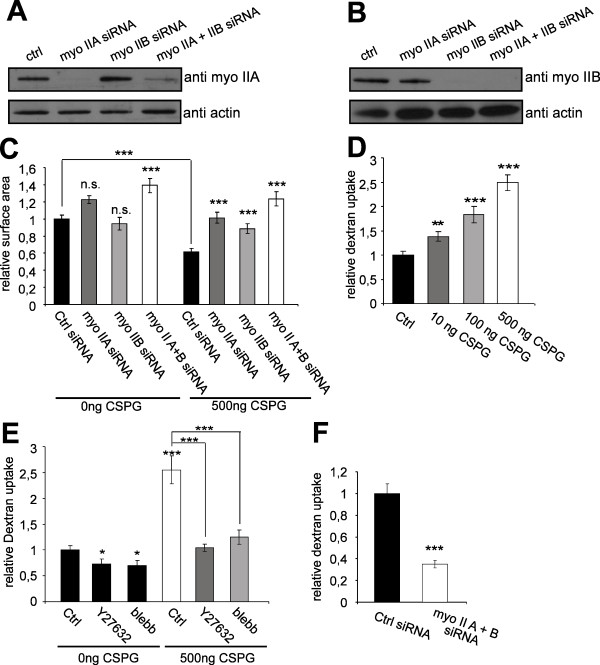
**Myosin II regulates cell surface area and endocytosis in Oli-neu cells**. (A+B) Western blot analysis of myosin (myo) IIA and IIB after siRNA knock down in Oli-neu cells as compared to actin as a loading control. (C) Control siRNA, siRNA directed against myosin (myo) IIA, myosin (myo) IIB or both were nucleofected into Oli-neu cells. The cells were subsequently cultured on CSPG coated coverslips and changes in cell surface area were quantified after 16 h (means ± SEM; n > 100 cells, n.s. not significant, ***p < 0,001). (D) Quantification of dextran uptake (30 min) in Oli-neu cells cultured on CSPG coated coverslips (means ± SEM; n > 100 cells, **p < 0,01, ***p < 0,001). (E) Quantification of dextran endocytosis in Oli-neu cells cultured on CSPG coated or control coverslips and treated with 10 μM Y27632 or 50 μM blebbistatin for 1 h (means ± SEM; n > 80 cells, *p < 0,05,***p < 0,001). (F) Quantitative analysis of the endocytosis of dextran in Oli-neu cells nucleofected with control siRNA or siRNA against both myosin IIA and IIB (means ± SEM; n > 80 cells from 2 independent experiments, ***p < 0,001). Scale bar 20 μm.

Blebbistatin is a small molecule inhibitor with high affinity and selectivity towards myosin II [26]. To confirm the role of myosin IIA and/or IIB we used a siRNA knock-down approach. Western blot analysis demonstrated the efficient knock-down of both myosin IIA (Figure 5A) and myosin IIB (Figure 5B). Knock-down of myosin IIA and IIB promoted cell surface extension on CSPG. The strongest growth promoting effects were observed when both myosins were reduced at the same time (Figure 5C).

To analyze whether the changes in cell surface area again correlate with changes in fluid-phase endocytosis, we analyzed the rate of dextran uptake in Oli-neu cells cultured on CSPG. We found that dextran was taken up more efficiently with increasing concentrations of CSPG (Figure 5D). When cells were treated with either blebbistatin or Y27632, the increase in fluid-phase endocytosis by CSPG was completely prevented (Figure 5E). Furthermore, we observed that the combined knock-down of myosin IIA and IIB reduced fluid-phase uptake (Figure 5F).

### Inhibition of actomyosin contractility promotes spreading of myelin-membrane sheets on a non-permissive substrate

Next, we used primary cultures of oligodendrocytes for our experiments. These cells differentiate within a few days from cells with multiple branches to myelin-membrane sheet producing cells. In cell culture the differentiation of oligodendrocytes follows a default pathway and occurs in the absence of extrinsic signals from neuronal cells.

When primary cells were treated with blebbistatin or Y27632 cell surface area did not change significantly. We analyzed whether growth on a non-permissive substrate suppresses myelin-membrane extension and if so, whether inhibition of actomyosin contractility could overcome the block.

**Figure 6:**
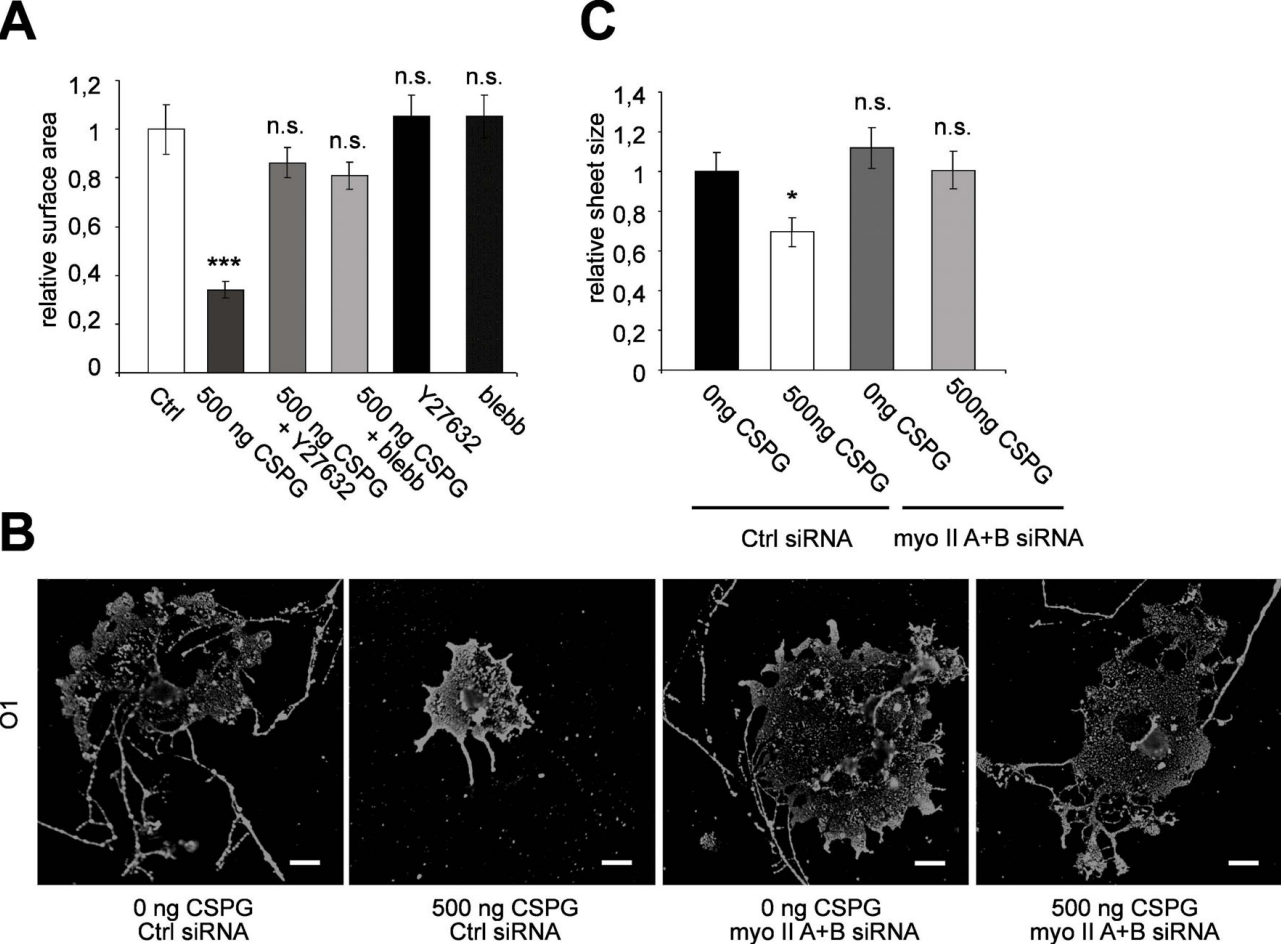
**Inhibition of Myosin II promotes spreading of myelin-membrane sheets on CSPG**. (A) Primary oligodendrocytes were cultured on CSPG coated or control coverslips treated with 10 μM Y27632 or 50 μM blebbistatin 2 h after seeding, stained for MBP and analyzed for changes in the size of myelin membrane sheets. Changes in cell surface area were quantified after 2-3 days. Values represent means ± SEM (n > 60 cells, n.s. not significant, ***p < 0,001). (B, C) Primary oligodendrocytes were nucleofected with siRNA directed against both myosin (myo) IIA and IIB or control (Ctrl) siRNA. The cells were subsequently cultured on CSPG coated or control coverslips and membrane sheet size was analyzed after 3 d. (C) Quantitative analysis of membrane sheet size (means ± SEM; n > 30 cells, *p < 0,05). (B) Primary oligodendrocytes stained with O1 antibody to visualize membrane sheets. Scale bar 20 μm.

When primary oligodendrocytes were cultured on 500 ng CSPG for 3 days (50 000 cells/3.8 cm^2^) there was a significant inhibition of membrane sheet extension (Figure 6A). The effect of the CSPG peptide was not observed to the same extent when the cells were cultured at higher density, indicating that unknown growth factors when present in sufficient amounts are able to overcome the inhibitory cues.

Next, we analyzed whether treatment with inhibitors to block actomyosin contractility promoted the spreading of membrane sheets on CSPG coated coverslips. Indeed, inhibitors of the Rho/ROCK pathway and myosin II were able to almost completely overcome the inhibition of cell surface expansion induced by CSPG (Figure 6A).

To confirm the role of myosin IIA and/or IIB we performed siRNA knock-down experiments in primary cells. Knock-down of myosin IIA and IIB promoted cell surface extension on CSPG as observed for Oli-neu cells (Figure 6B, C). Together, these results show that the reduction of myosin II activity is sufficient to overcome the non-permissive substrate induced inhibition of plasma membrane growth.

## Discussion

In this study, we show that cell surface area of oligodendrocytes is critically dependent on actomyosin contractility and is regulated by the physical properties of the supporting matrix. Furthermore, we find that chondroitin sulfate proteoglycans (CSPG), molecules associated with non-permissive growth properties within the central nervous system (CNS), block cell surface spreading. In addition, we observed that the reductions of plasma membrane area were accompanied by changes in the rate of fluid-phase endocytosis. We found that this form of endocytosis is dependent on actomyosin contractility and possibly also on integrins. A possible explanation is that actomyosin contractility pulls membrane into the cell and thereby accelerates vesicle budding. Loss of integrin-mediated anchoring to the extracellular matrix may increase these pulling forces of the actin cytoskeleton. We show that this mechanism is important for cell surface spreading of Oli-neu cells, but it is likely to be involved in the control of cell surface area in other systems. It may be particularly relevant in processes where rapid changes of cell surface area occur, for example in a blood vessel or a bladder [27].

Previous work shows that extracellular matrix proteins, like laminin, promote the formation of myelin membrane sheets in oligodendrocytes by its interaction with integrin α6β1 [28,29]. Oligodendrocytes are known to express a large number of additional integrins including αvβ1, αvβ3 and α5β5, which may regulate different aspects of oligodendrocyte biology such as proliferation, differentiation and survival [9,30-34].

In addition to signals from the extracellular matrix, paracrine signals from oligodendrocytes may also regulate myelin-membrane extension. Interestingly, the effects of the CSPG peptide on oligodendrocyte cell surface area was not observed when cells were grown at a high density, indicating oligodendrocyte present factors to neighboring cells that are able to overcome the inhibitory cues.

Integrin-mediated cell attachment is known to regulate endocytosis and cell surface shape. A striking example of the importance of this mechanism is the loss of integrin-mediated adhesion during tumor growth, which triggers rapid endocytosis and cell rounding [1]. The growth promoting properties of extracellular matrix proteins in oligodendrocytes might thus in part be explained by integrin-mediated retention of membrane at the cell surface. In addition, a role of extracellular matrix proteins in regulating vesicular traffic within the biosynthetic pathway to the myelin membrane sheets has been proposed [35].

The finding that actomyosin contractility acts downstream of integrins is consistent with a report describing a signal transduction pathway from integrins to Fyn that facilitates RhoA GTPase inactivation and the morphological differentiation of oligodendrocytes [10]. Our results extend this pathway by providing evidence that myosin II is a downstream target that is regulated by integrins and Rho/ROCK activity. This concurs with the finding that myosin II inactivation promotes myelination in the CNS [36].

In the adult brain OPCs are kept in an undifferentiated state. This may in part be due to the presence of myelin from mature oligodendrocytes that blocks their differentiation [37]. Whether this occurs by Rho/ROCK/myosin II activation is an interesting question for future research. After demyelination OPCs are recruited to the site of the lesion where they differentiate. In multiple sclerosis remyelination can be extensive, however, OPC frequently fail to differentiate [38-41]. The inhibitory cues for OPCs in multiple sclerosis lesions have not been identified, but upregulation of growth inhibiting molecules such as CSPG within the glial scar is one possibility.

We do not know by what means CSPGs affects cell surface size, but the finding that knocking down myosin II overcomes CSPGs inhibitory effects points to a role of actomyosin contractility in this process. Earlier work examining the role of CSPGs on axon outgrowth suggested that CSPGs interfere with extracellular matrix/integrin interactions [42].

Indeed, increased integrin expression is sufficient to eliminate the inhibitory effects of CSPGs on neuronal growth cone function [43]. A down-stream role of the Rho/ROCK signalling system has also been identified [25].

Our results demonstrating that inhibition of Rho/ROCK/myosin II activity is able to overcome a non-permissive growth property of the extracellular environment on oligodendrocytes extends the role of this system in cellular regeneration.

These findings not only show that the actomyosin system plays a role in cell surface spreading of oligodendrocytes, but also point to potential target for therapeutic interventions. However, we do not yet know whether the inhibition of actomyosin contractility affects the ability of oligodendrocytes to wrap their plasma membrane around an axon to form compact myelin. Clearly, in vivo studies are now required to determine whether inhibition of actomyosin contractility promotes myelin repair in the CNS.

## Conclusion

In summary, we find that cell surface area depends on actomyosin contractility and is regulated by physical properties of the supporting matrix. CSPG blocks cell surface spreading, but the inhibitory effect of CSPG on plasma membrane extension is prevented by treatment with inhibitors of actomyosin contractility and by RNAi mediated knockdown of myosin II. These results establish a novel connection between cell surface extension and actomyosin contractility in oligodendrocytes and open up new possibilities of how to promote the morphological differentiation of oligodendrocytes in a non-permissive growth environment.

## Authors' contributions

AK performed the experiments, analyzed the data, drafted the manuscript; JH performed the atomic force microscopy measurements; DF performed experiments, analyzed the data and approved the final manuscript; MS designed and coordinated the work and wrote the manuscript. All authors read and approved the final manuscript.
